# Attitudes and perceptions of Thai medical students regarding artificial intelligence in radiology and medicine

**DOI:** 10.1186/s12909-024-06150-2

**Published:** 2024-10-22

**Authors:** Salita Angkurawaranon, Nakarin Inmutto, Kittipitch Bannangkoon, Surapat Wonghan, Thanawat Kham-ai, Porched Khumma, Kanvijit Daengpisut, Phattanun Thabarsa, Chaisiri Angkurawaranon

**Affiliations:** 1 Department of Radiology, Faculty of Medicine, Chiang Mai University, Chiang Mai, Thailand; 2 Global Health and Chronic conditions Research Center, Chiang Mai University, Chiang Mai, Thailand; 3 Department of Radiology, Faculty of Medicine, Prince of Songkla University, Songkla, Thailand; 4 Faculty of Medicine, Chiang Mai University, Chiang Mai, Thailand; 5 Department of Family Medicine, Faculty of Medicine, Chiang Mai University, Chiang Mai, Thailand

**Keywords:** Artificial intelligence, Medical students, Radiology and medicine, Thailand, Attitudes and perceptions

## Abstract

**Introduction:**

Artificial Intelligence (AI) has made a profound impact on the medical sector, particularly in radiology. The integration of AI knowledge into medical education is essential to equip future healthcare professionals with the skills needed to effectively leverage these advancements in their practices. Despite its significance, many medical schools have yet to incorporate AI into their curricula. This study aims to assess the attitudes of medical students in Thailand toward AI and its application in radiology, with the objective of better planning for its inclusion.

**Methods:**

Between February and June 2022, we conducted a survey in two Thai medical schools: Chiang Mai University in Northern Thailand and Prince of Songkla University in Southern Thailand. We employed 5-point Likert scale questions (ranging from strongly agree to strongly disagree) to evaluate students’ opinions on three main aspects: (1) their understanding of AI, (2) the inclusion of AI in their medical education, and (3) the potential impact of AI on medicine and radiology.

**Results:**

Our findings revealed that merely 31% of medical students perceived to have a basic understanding of AI. Nevertheless, nearly all students (93.6%) recognized the value of AI training for their careers and strongly advocated for its inclusion in the medical school curriculum. Furthermore, those students who had a better understanding of AI were more likely to believe that AI would revolutionize the field of radiology (*p* = 0.02), making it more captivating and impactful (*p* = 0.04).

**Conclusion:**

Our study highlights a noticeable gap in the understanding of AI among medical students in Thailand and its practical applications in healthcare. However, the overwhelming consensus among these students is their readiness to embrace the incorporation of AI training into their medical education. This enthusiasm holds the promise of enhancing AI adoption, ultimately leading to an improvement in the standard of healthcare services in Thailand, aligning with the country’s healthcare vision.

**Supplementary Information:**

The online version contains supplementary material available at 10.1186/s12909-024-06150-2.

## Introduction

Currently, Artificial Intelligence (AI) has made a significant impact in the medical sector, particularly in the field of radiology. The clinical applications of AI are vast, ranging from augmenting patient care through improving the prediction, diagnostic, and prognostic process [1] to other administrative aspects such as creating discharge summaries [2, 3]. In radiology, AI has been useful in helping the interpretation of breast imaging, chest X-rays, and CT scans [4, 5]. AI can also help to improve workflow by creating hanging protocols and helping schedule scanners and patients [5].

Its assistance has proven to be invaluable in diagnosis, such as identifying pulmonary tuberculosis through chest radiography [6–8] and determining the location of intracranial hemorrhage in patients who have suffered traumatic brain injuries through computed tomography scans [9, 10]. There are over 150 AI products for radiology on the market [11]. These products have been approved by the Food and Drug Administration or CE-marked to allow clinical use in the United States and Europe. With growing academic and industry interest, validated use cases for AI tools in radiology are expected to emerge quickly. It is imperative that medical students are adequately prepared for the era of AI. Knowledge and understanding of AI need to be integrated into medical education to ensure that future healthcare professionals can effectively utilize these advancements in their practices. However, many medical schools in developed countries have introduced and integrated concepts of AI in medical education [12], many developing countries are in early stages of incorporate AI into their medical school curricula [13–16].

The lack of proposer understanding of AI, particularly in its application in radiology, is reflected in the literature. A study from Germany in 2018 shows most medical students do not comprehend the core technical ideas underlying AI [17]. In one study among radiologists, surgeons, and students, the majority agreed that AI could support radiological image evaluations. However, in the same study, 26% of students who did not want to pursue a career in radiography cited AI as one of their reasons [18]. Likewise, a Canadian study discovered that most medical students hesitate to pursue a career in radiography because they fear being replaced by AI [19]. In contrast, a German survey indicated that only 17% of medical students believe AI will replace them [17], while a study from the United Kingdom found that the number of medical students who believed AI would replace some medical fields (49%) is roughly the same as who did not (51%) [20]. However, most studies were conducted in developed countries, and a recent review suggested that insights from developing countries are still required [16, 21].

Like many countries, Thailand aims to improve health service standards through AI and advanced technology [22]. To achieve this, familiarizing medical students with AI concepts and ideas is of utmost importance. This will enable them to incorporate AI into patient care effectively, avoiding obstacles caused by complex terminology and definitions. The aim of this study is to examine medical student’s perception of (1) basic understanding of AI, (2) teaching of AI in medical school, and (3) the impact of AI on medicine and radiology. Understanding medical students’ awareness and learning needs regarding AI will be beneficial in planning and delivering effective and relevant teaching strategies to meet their needs [23]. Moreover, in an era where AI is becoming more prevalent, medical student’s attitudes toward Radiology may be influenced by their ignorance of AI. The shortage of radiologists is a major concern, not just in the United States or the United Kingdom but in many countries worldwide [24]. Awareness of medical students’ attitudes towards AI and its application in radiology may lead to better planning and preparedness in addressing any potential challenges.

## Materials and methods

A survey was conducted between February and June 2022 in two medical schools in Thailand: Chiang Mai University in Northern Thailand and Prince of Songkla University in Southern Thailand. Based on a prior study from Singapore [25], it was estimated that at least 331 participants were required. A Google form questionnaire was distributed online through email and social media platforms to medical students in all years of the medical education program, from the first to the sixth year.

Informed consent was obtained from all participants. Ethics approval was obtained from Faculty of Medicine, Chiang Mai University Ethical Committee (approval number 039/2022) and from Faculty of Medicine, Prince of Songkla Ethical Committee (reference number 65-099-701).

Demographic data on age, sex, and year of medical school was collected. In Thailand, the first three years of medical school are considered pre-clinical years, and the last three years are considered clinical years. The questionnaire regarding perceptions of AI in this study was based on a prior multicenter study by Sit C. et al. regarding UK medical students’ attitudes towards AI and radiology [20] with some additional questions based on similar research by Pinto D. et al. [17], Gong B. et al. [19] and Wood EA. et al. [26]. The investigators discussed these items and came to a consensus on which items would be most appropriate for the study, keeping in mind the local Thai settings as well as the potential values for cross-cultural comparisons. The development of the Thailand questionnaire followed the WHO process of translation and adaptation of instrument protocol [27]. This included forward and backward translation, content validity was approved by local experts, and the questionnaire was piloted on ten medical students. 5-point Likert questions (strongly agree, agree, neutral, disagree, and strongly disagree) were used to assess the students’ agreement towards three main aspects: (1) their understanding of AI, (2) teaching of AI, and (3) the impact of AI on medicine and radiology. (Appendix 1). For their basic understanding of AI, students were asked about their prior exposure to training and their understanding of AI’s underlying principles and limitations. For teaching of AI, students were asked about their expectations of AI teaching, potential topics of interest, and preferred learning methods. Lastly, students were asked about their perceptions of the potential impact of AI in medicine and radiology.

Using frequency and percentages, descriptive analysis was used to describe the medical student’s perception of AI. The 5-point scale was simplified into a binary variable combining those who answered “strongly agree” and “agree” into one group and those who answered “neutral,” “disagree,” or “strongly disagree” into another group. To explore potential differences in perceptions of the impact of AI on Radiology between those who have a basic understanding of AI and those who do not, chi-square was used. Analyzes were performed using STATA 15, and a p-value of < 0.05 was considered statistically significant.

## Results

A total of 352 medical students across the six years of medical school responded (Table 1). The overall response rate was 13.6%, varying between 11 and 18% across different years between the two intuitions (appendix 2). One hundred ninety-nine respondents were from Chiang Mai University (56.5%), and 153 were from Prince of Songkla University (43.5%). One hundred sixty-five respondents are male (46.9%),181 respondents are female (51.4%), and six respondents did not indicate their gender (1.7%). The mean age of the respondents was 21.8 years (sd = 1.8). There were no missing values in the survey responses.

**Table 1:** Characteristics of the respondents

Demographics	Total Participants (*n* = 352)	Chiang Mai University (*n* = 199)	Prince of Songkla University (*n* = 153)
Age in year (sd)	21.8 (1.84)	21.3 (1.9)	21.6 (1.8)
Gender (n, col%)			
Male	165 (46.9)	90 (45.2)	75 (49.0)
Female	181 (51.4)	106 (53.3)	75 (49.0)
not specified	6 (1.7)	3 (1.5)	3 (2.0)
Year in Medical School (n, col%)			
1	59 (16.8)	27 (13.4)	32 (20.9)
2	60 (17.0)	37 (18.6)	23 (15.0)
3	91 (25.9)	62 (31.2)	29 (19.0)
4	60 (17.0)	31 (15.6)	29 (19.0)
5	43 (12.2)	24 (12.1)	19 (12.4)
6	39 (11.1)	18 (9.1)	21 (13.7)

### Basic understanding of artificial intelligence

For the background of AI exposure, the minority of respondents (29.3%, *n* = 103) said they had studied artificial intelligence. The majority (77.7) of those with prior AI training came from pre-clinical students (years 1–3). About 31% of participants felt that they understood basic computational principles, and 43.2% thought that they understood the limitations of AI (Table 2). These two perceptions did not significantly differ between students in pre-clinical years and those in their clinical year, obtaining a p-value of 0.58 and 0.13, respectively.

**Table 2:** Basic understanding of Artificial Intelligence

	Strongly agree	Agree	Neutral	Disagree	Strongly disagree
**Basic understanding (n, row%)**					
I have an understanding of the basic computational principles of artificial intelligence	31 (8.8)	80 (22.7)	129 (36.6)	84 (23.9)	28 (7.9)
I have an understanding of the limitations of artificial intelligence.	47 (13.4)	105 (29.8)	119 (33.8)	64 (18.2)	17 (4.8)
I consider myself a tech-savvy person	19 (5.4)	55 (15.6)	153 (43.4)	95 (27.38)	29 (8.36)

### Teaching of AI in medical school

The vast majority of students felt that teaching AI would benefit their careers (88.5%) and that all medical students should receive training in AI (76.9%). The majority of students (> 75%) agreed that common topics such as “AI in clinical application”, “Basic AI principal”, “AI in Radiology”, “AI in diagnostic and clinical decision support”, and “Ethics and laws related to AI” should be taught in medical school (Table 3). As for their current needs, about 58% of medical students would like a list of resources where they can learn about AI, 55% would like to hear experts discuss the impact and application of AI, and 48% would like the medical school to offer courses on AI.

**Table 3:** Medical students attitude towards teaching of AI

	Strongly agree	Agree	Neutral	Disagree	Strongly disagree
**General perception (n, row %)**					
Teaching in artificial intelligence will be beneficial for my career	191 (54.2)	121 (34.4)	34 (9.7)	5 (1.4)	1 (0.3)
All medical students should receive teaching in artificial intelligence.	150 (42.6)	121 (34.4)	59 (16.8)	21 (6.0)	1 (0.3)
Topics to be taught in medical school (n, row %*)					
Basic AI principal	149 (42.9)	118 (34.0)	67 (19.3)	11 (3.2)	2 (0.6)
AI in clinical applications	197 (56.8)	117 (33.7)	25 (7.2)	4 (1.1)	4 (1.1)
AI in Radiology	140 (40.3)	142 (40.9)	55 (15.9)	7 (2.0)	3 (0.9)
AI in Disease Prediction models	131 (37.7)	156 (45.0)	51 (14.7)	5 (1.4)	4 (1.1)
AI in medical genetics and genomics	141 (40.6)	120 (34.6)	66 (19.0)	16 (4.6)	4 (1.1)
AI diagnostic and clinical decision support	141 (40.6)	118 (34.0)	66 (19.0)	16 (4.6)	6 (1.7)
Ethics and laws related to AI	155 (44.7)	106 (30.6)	57 (16.4)	24 (6.9)	5 (1.4)

* missing data = 5

### Impact of AI on medicine and radiology

Nearly all medical students (93.6%) agreed that AI will play an important role in health care, and 45% agreed that AI may replace some specialty in their lifetime. Moreover, 82% felt that AI could revolutionize radiology. However, only 38% felt that AI makes radiology more exciting, and about 28% thought that they were less likely to consider a career in radiology given the advancement of AI (Table 4).

**Table 4:** Medical students’ attitude towards AI in Medicine and Radiology

	Strongly agree	Agree	Neutral	Disagree	Strongly disagree
**Impact of AI in Medicine (n, row %)**					
Artificial intelligence will play an important role in healthcare.	216 (61.4)	114 (32.4)	21 (6.0)	1 (0.3)	0 (0.00)
Some specialties will be replaced by artificial intelligence during my lifetime	69 (19.6)	90 (25.6)	110 (31.3)	60 (17.1)	23 (6.5)
**Impact of AI in Radiology (n, row%)**					
Artificial intelligence will revolutionize radiology	134 (38.1)	156 (44.3)	54 (15.3)	8 (2.3)	0 (0.00)
Artificial intelligence makes radiology more exciting for me	50 (14.2)	83 (23.6)	131 (37.75)	50 (14.41)	35 (10.09)
I am less likely to consider a career in radiology, given the advancement of artificial intelligence.	38 (10.8)	63 (17.9)	97 (27.6)	95 (27.0)	59 (16.8)

**Fig. 1:**
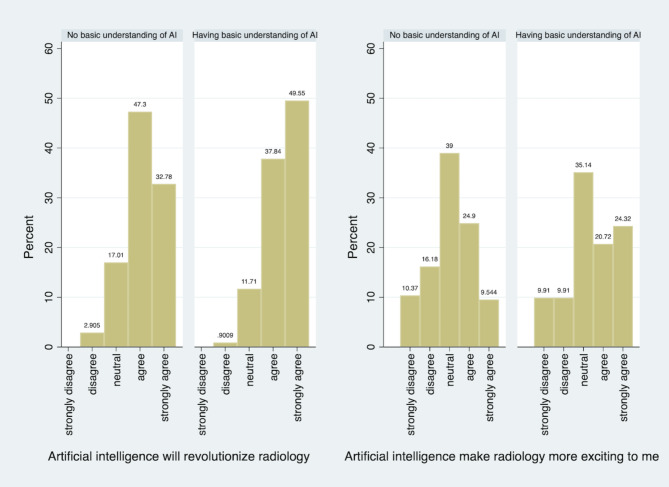
Perceptions of Impact on Radiology by level of basic understanding of AI

The perception of the impact of AI on medicine and radiology differed between those who had a basic understanding of AI (Fig. 1). Those with a basic understanding were more likely to feel that AI will play an important role in health care (*p* = 0.04), revolutionize radiology (*p* = 0.02), and make radiology more exciting (*p* < 0.01). There were no significant differences between pre-clinical and clinical years (*p* > 0.05 in all subgroup analysis).

## Discussion

The multicenter study explored medical student’s perception of basic understanding of AI, training needs, and the impact of AI on medicine and radiology. Currently, only one-third of medical students have a basic understanding of AI. Almost all students perceived that training on AI would benefit their careers, should be taught in medical school and will play an important role in health care. Those who understand AI were more likely to agree that AI will revolutionize radiology and make radiology more exciting for them.

The vast majority of respondents anticipated that AI would play a significant part in healthcare in the coming years, just like the student from other countries [16, 17, 20, 26, 28, 29]. This emphasizes that the awareness of the impact of AI is not limited to Western countries. Meanwhile, medical students self-reported varying levels of expertise in AI [16, 28]. Of concern, however, is the low proportion of students with knowledge of AI’s foundational ideas and concepts, which is in the line with previous research from the United States of America, United Kingdom [20, 25, 26, 30]. This indicates that AI education is still lacking in medical school. In our study, only one-third of students have studies or been exposed to artificial intelligence. In our survey, barely one-third of students had studied or experience AI. However, this study collected data prior to the November 2022 debut of ChatGPT, a user-friendly large language models (LLMs) that has rapidly gained popularity. Since ChatGPT’s release, there has been a significant increase in publications and interest in AI, particularly in the medical field and medical education [16, 31]. ChatGPT may have been used as a personalized learning assistant by both students and teachers [31]. Given ChatGPT’s growing role as a tool with great potential to assist undergraduate medical students [32],  it is likely that students’ exposure may lead to a shift in perceptions. A recent survey from UAE published in 2023 suggest that 20% have used ChatGPT in medical school and were optimistic about the positive impact of AI in medicine [37]. It is also reflected in a 2024 study which found that only 6% of radiology trainees and radiologist felt that AI proposes a threat to radiology job market [24].

The study reflects that medical students in Thailand support that common topics such as “AI in clinical application,” “Basic AI principal,” and “Ethics and laws related to AI” should be taught in medical school. Literature suggests that curricular components should be tailored to meet physicians’ daily practice, while strong extracurricular programs can be tailored to encourage innovation [33]. As for preferred learning methods, Thai medical students would like to attend courses on AI, access a list of resources to learn about AI and attend expert discussion forums.

There have been calls to adopt teaching on AI in undergraduate medical education [34]. The teaching of AI in medical education is rapidly evolving, with several pilot training being introduced in many countries [34]. There are also extensive courses related to AI in clinical practice for physicians. These courses are offered for continuing medical education for physicians in the United States [35]. However, faculty resistance, limited curricular hours, and lack of AI core competencies are known barriers to integrating AI training into the medical curriculum [34].

Radiology is one discipline where AI should significantly impact current practice [18]. Most respondents believe AI will revolutionize radiology, which is an excellent starting point. Just one-third of our respondents think that AI makes radiology more interesting, similar to a prior European study [17]. About 28% of Thai medical students agreed that a career in radiology is less likely to be considered, given the development of artificial intelligence. The result is lower than that of a previous study conducted in the United Kingdom in 2020, which found that roughly half of the students concurred [20]. In contrast, a 2024 study by Hassankhani et al. [29] found that only 20.3% of participants agreed that advancements in AI technology would diminish their inclination to pursue a profession in radiology. Earlier debates on the role of AI in radiology evolved around the theme that “AI will replace the radiologist.” However, in recent years, the understanding of the role of AI in radiology has shifted to “Radiologists who use AI will replace radiologists who do not” [36, 37]. Demonstrating this shift in the role of AI in radiology, recent research has concentrated on how AI may complement radiologists’ performance rather than testing the performance of AI against the other [37–39].

In our study, we found some relation between the understanding of the basic principles of AI and the consideration that AI makes radiology more exciting. This is similar to a previous study, which showed that tech-savvy students tended to be more positive about how AI will enhance medicine [17]. Sit et al. also discovered that students who received AI instruction were significantly less likely to deny radiology as a prospective career field [20]. From these results, it can be implied that the students who understand the basics of AI may have a more positive attitude toward the field of Radiology than those who do not. Staff shortages in clinical radiology are escalating annually, delaying patient diagnoses and negatively affecting patient care [40, 41]. These findings provide additional support for the urgent implementation of AI in medical education.

There are strengths and weaknesses to the study. This is one of the first multicenter surveys of medical student’s perceptions of AI in Thailand. It achieved a larger sample size than other recent studies from Germany [17], Singapore [25] and Canada [19]. However, the main weakness of our study are we used non-probability sampling from two Thai medical institutions with modest response rates which may not be a representative sample of Thai medical students and limit generalization. However, studies have suggested that there may not be a direct link between lower response rate and validity [42]. Additionally, like other published literature using online surveys, there may be a risk of self-selection bias among respondents because only medical students who were interested in employing AI in medicine voluntarily participated in the survey. If so, it would likely overestimate the actual proportional of students in Thailand who are enthusiastic or have some basic understanding of AI. To increase generalizability, future studies should aim for more representation, higher response rate, and use of systematic sampling. Lastly, this study took place before the introduction of LLMs, particularly Chat GPT, officially released on November 30, 2022. Studies have identified the impact of large language models (LLMs) in medical education ranging from helping with medical decision making, preparing for exams and academic writing [32, 43, 44]. A consistent theme exists that further research is needed to explore the impact of AI in medical education and how the shift of perception and use of AI differ after the introduction of LLMs.

## Conclusion

In contrast to the continuously increasing use of AI in medicine and radiology, there is a gap in medical students’ understanding of AI and its practical applications. However, an overwhelming number of students are aware of the potential and benefits of AI in healthcare. The majority of medical students in Thailand seem ready to embrace the change in their medical curricula to include training in AI. Proper undergraduate training may help enhance AI adoption to improve the standard of health services as envisioned in Thailand.

## Electronic supplementary material

Below is the link to the electronic supplementary material.

Supplementary Material 1

Supplementary Material 2

## Data Availability

The datasets used and/or analysed during the current study are available from the corresponding author on reasonable request.

## Abbreviations

AI: Artificial Intelligence
