# Supplementary material for: Attitudes and perceptions of Thai medical students regarding artificial intelligence in radiology and medicine
Source: BMC Med Educ. 2024 Oct 22;24:1188. doi: 10.1186/s12909-024-06150-2 (PMC11515691; doi:10.1186/s12909-024-06150-2)
Supplement: Supplementary file 1 — Supplementary Material 1 [file 12909_2024_6150_MOESM1_ESM.docx]

**Appendix 1**

| **Demography** | | | | | |
| --- | --- | --- | --- | --- | --- |
| Age | ____ years | | | | |
| Gender | □ Male  □ female  □ no response | | | | |
| You are a medical student at | □ Chiang Mai University  □ Prince of Songkla University | | | | |
| Your year level is | □ First year  □ Second year  □ Third year  □ Fourth year  □ Fifth year  □ Sixth year | | | | |
| **Section:** Basic understanding of artificial intelligence (AI) | | | | | |
|  | Strongly agree | Agree | Neither agree or disagree | Disagree | Strongly disagree |
| I have an understanding of the basic principles of AI | □ | □ | □ | □ | □ |
| I have an understanding of the limitations of AI | □ | □ | □ | □ | □ |
| I have received teaching/training in artificial intelligence | □ Yes  □ No | | | | |
| **Section:** Teaching of artificial intelligence in Medical School | | | | | |
|  | Strongly agree | Agree | Neither agree or disagree | Disagree | Strongly disagree |
| Teaching in artificial intelligence will be beneficial for my career | □ | □ | □ | □ | □ |
| All medical students should receive teaching in artificial intelligence | □ | □ | □ | □ | □ |
| In your opinion, what can we do to help medical students make informed specialty decision regarding the impact of artificial intelligence on radiology? | Please choose the most important initiatives. Select up to 3.  □ Offer courses on artificial intelligence.  □ Provide a list of resources about artificial intelligence.  □ Invite experts to provide opinions on the impact of artificial intelligence.  □ Discuss artificial intelligence in radiology lectures.  □ Create radiology research projects involving artificial intelligence.  □ Other | | | | |
|  | Very unimportant | Unimportant | Neither important or unimportant | Important | Very important |
| How important, in your opinion, are the following AI topics the education of medical students and/or residents?  - Basic artificial intelligence knowledge  - Clinical applications  - Radiology  - Disease prediction models  - Medical genetics and genomics  - Diagnostics and clinical decision support  - Law and ethics related to artificial intelligence | □  □  □  □  □  □  □ | □  □  □  □  □  □  □ | □  □  □  □  □  □  □ | □  □  □  □  □  □  □ | □  □  □  □  □  □  □ |
| **Section:** Impact of AI on Medicine and Radiology | | | | | |
|  | Strongly agree | Agree | Neither agree or disagree | Disagree | Strongly disagree |
| AI will play an important role in healthcare | □ | □ | □ | □ | □ |
| Artificial intelligence will revolutionize radiology | □ | □ | □ | □ | □ |
| Artificial intelligence makes radiology more exciting to me | □ | □ | □ | □ | □ |
| I am LESS likely to consider a career in radiology, given the advancement of AI | □ | □ | □ | □ | □ |
