# Supplementary material for: Attitudes and perceptions of Thai medical students regarding artificial intelligence in radiology and medicine
Source: BMC Med Educ. 2024 Oct 22;24:1188. doi: 10.1186/s12909-024-06150-2 (PMC11515691; doi:10.1186/s12909-024-06150-2)
Supplement: Supplementary file 2 — Supplementary Material 2 [file 12909_2024_6150_MOESM2_ESM.docx]

**Appendix 2 Response rates**

| Year in Medical School | Chiang Mai University | | | Prince of Songkla University | | |
| --- | --- | --- | --- | --- | --- | --- |
|  | Respondents  (n) | Total Students (n) | Response Rate (%) | Respondents  (n) | Total Students (n) | Response Rate (%) |
| 1 | 35 | 253 | 13.8 | 32 | 192 | 16.7 |
| 2 | 35 | 263 | 13.3 | 23 | 182 | 12.6 |
| 3 | 43 | 229 | 18.8 | 29 | 187 | 15.5 |
| 4 | 31 | 232 | 13.4 | 29 | 194 | 14.9 |
| 5 | 28 | 245 | 11.4 | 19 | 189 | 10.1 |
| 6 | 27 | 236 | 11.4 | 21 | 178 | 11.8 |
| Overall | **199** | **1,458** | **13.6** | **153** | **1,122** | **13.6** |
